# Identification of *cbiO* Gene Critical for Biofilm Formation by MRSA CFSa36 Strain Isolated from Pediatric Patient with Cystic Fibrosis

**DOI:** 10.3390/pathogens10111363

**Published:** 2021-10-21

**Authors:** Ying Liu, Junshu Yang, Michelle Ji, James Phillips, Mark Wylam, Yinduo Ji

**Affiliations:** 1 Department of Veterinary Biomedical Sciences, College of Veterinary Medicine, University of Minnesota, Saint Paul, MN 55108, USA; liuyd@shafc.edu.cn (Y.L.); yang1181@umn.edu (J.Y.); 2 Department of Biomedicine and Health Sciences, Shanghai Vocational College of Agriculture and Forestry, Shanghai 201699, China; 3 School of Medicine, Creighton University, Omaha, NE 68178, USA; MichelleJi@creighton.edu; 4 Division of Pulmonary and Critical Care Medicine, Mayo Clinic College of Medicine, Rochester, MN 55905, USA; James.R.Phillips@gunet.georgetown.edu (J.P.); Wylam.Mark@mayo.edu (M.W.); 5 Division of Pulmonary and Critical Care Medicine, MedStar Georgetown University Hospital, Washington, DC 20007, USA

**Keywords:** *Staphylococcus aureus*, MRSA, biofilm formation, *cbiO*, copper ions

## Abstract

The colonization of *Staphylococcus aureus*, especially methicillin-resistant *S. aureus* (MRSA), has a detrimental effect on the respiratory care of pediatric patients with cystic fibrosis (CF). In addition to being resistant to multiple antibiotics, *S. aureus* also has the ability to form biofilms, which makes the infection more difficult to treat and eradicate. In this study, we examined the ability of *S. aureus* strains isolated from pediatric patients with CF to form biofilms. We screened a transposon mutant library of MRSA and identified a putative cobalt transporter ATP binding domain (*cbiO*) that is required for biofilm formation. We discovered that deleting *cbiO* creating a *cbiO* null mutant in CFSa36 (an MRSA strain isolated from a patient with cystic fibrosis) significantly hinders the ability of CFSa36 to form biofilm. The complementation of *cbiO* restored the ability of the *cbiO* deletion mutant to generate biofilm. Interestingly, we revealed that incorporating extra copper ions to the chemically defined medium (CDM) complemented the function of *cbiO* for biofilm formation in a dose-dependent manner, while the addition of extra iron ions in CDM enhanced the effect of *cbiO* null mutation on biofilm formation. In addition, neither the addition of certain extra amounts of copper ions nor iron ions in CDM had an impact on bacterial growth. Taken together, our findings suggest that *cbiO* mediates biofilm formation by affecting the transportation of copper ions in the MRSA CFSa36 strain. This study provides new insights into the molecular basis of biofilm formation by *S. aureus*.

## 1. Introduction

*Staphylococcus aureus* is an important opportunistic human pathogen that causes a variety of diseases. The growing prevalence of methicillin-resistant *S. aureus* (MRSA), including hospital-acquired MRSA (HA-MRSA) and community-acquired MRSA (CA-MRSA), has created a serious public health concern because most MRSA isolates are resistant to multiple antibiotics and result in poor patient outcomes. Thus, there is an urgent need to develop alternative therapeutic agents against MRSA.

MRSA is also a critical pathogen that causes infection in patients with cystic fibrosis (CF). CF is an autosomal recessive (AR) disorder caused by mutations in the cystic fibrosis transmembrane conductance regulator (CFTR). CF is the most common AR disorder in the western world, with an incidence of one case per 2500 live births [1]. Mutations in the CTFR gene result in defective chloride channels, which inhibit the flow of water across epithelial cell membranes and lead to hyperviscous secretions. In the respiratory tract, this viscous mucus impairs the mucociliary clearance of bacteria and predisposes patients to acute and persistent infections by opportunistic pathogens. Ultimately, chronic respiratory inflammation and infection in CF patients may progress to bronchiectasis and respiratory failure. *S. aureus* is often the first pathogen to rapidly colonize the airways of neonatal CF patients and occurs within days following birth [2]. Thus, preventing the initial airway injury from *S. aureus* may be central to CF respiratory care. CF patients who acquire pulmonary MRSA infections are particularly challenging to treat. Macrocolonies of MRSA are often found embedded within the mucinous layer and forming biofilms. This biofilm presence can clearly limit antibiotic killing due to (a) reduced antibiotic concentrations reaching bacteria due to the presence of polymerizable mucopolysaccharides on the biofilms, (b) entrapment of metabolically inactive bacteria within the biofilm, and (c) the accumulation of bacterial cells within the biofilm facilitatig horizontal gene transfer responsible for antibiotic resistence [3]. The ability of MRSA to form biofilms compounded with multidrug resistance significantly decreases the efficacy of standard drug therapy and leads to poor outcomes in CF patients [4].

The ability of *S. aureus* to form biofilms is dependent on varying gene expression as a response to its surrounding environmental conditions. Factors that affect biofilm formation include, but are not limited to, ambient oxygen tension, the presence of sub-inhibitory antibiotics, temperature, and pH [5,6]. Although biofilm can protect *S. aureus* viability, its maintenance requires a significant amount of energy and at certain thresholds, biofilm disassembly provides an outlet for enhanced survival and dissemination [5]. A major mechanism by which *S. aureus* degrades biofilm is through extracellular enzymes that solubilize the biofilm matrix. Studies have shown that exogenously added proteases such as tryptase, DNase, and restriction enzymes are capable of dismantling biofilms produced by *S. aureus* [5]. Therefore, targeting the genes that are critical for biofilm production and maintenance in *S. aureus* may provide an alternative approach to combat MRSA, especially in patients with CF.

In this study, we examined the ability of biofilm formation by *S. aureus* strains isolated from pediatric patients with CF and identified gene(s) critical for biofilm formation. We identified that a cobalt transporter ATP binding domain (*cbiO*) is important for biofilm formation by screening a Nebraska Transposon mutant library of CA-MRSA. We further determined the requirement of *cbiO* for biofilm formation by a MRSA strain CFSa36 by performing loss-of-function and gain-of-function studies. We created a defined *cbiO* deletion mutant of CFSa36 using homogeneous recombination and constructed a complementary strain. Moreover, we revealed that *cbiO* likely contributes to biofilm formation through mediating copper transportation. These findings provide new insight into the mechanisms of biofilm formation by MRSA.

## 2. Results

### 2.1. Characterization of the Capacity of Biofilm Formation by S. aureus Strains Isolated from Pediatric Patients with Cystic Fibrosis

**Figure 1:**
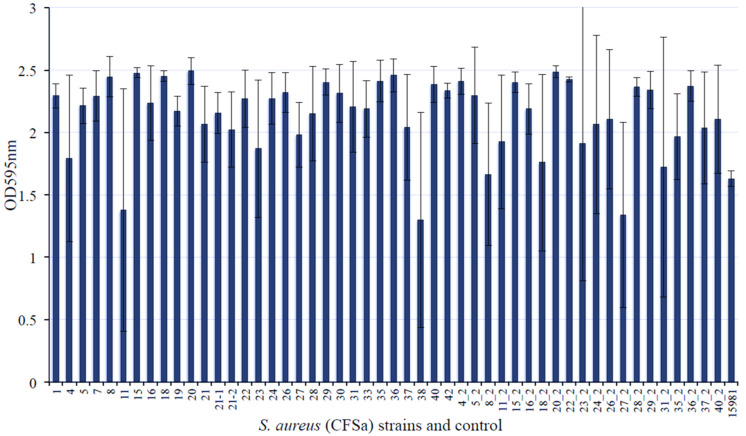
Comparison of biofilm formation by *S. aureus* strains isolated from the pediatric patients with cystic fibrosis. The bacterial strains were incubated in TSB supplemented with 3.0% NaCl and 0.5% glucose at 37 °C overnight with shaking at 220 rpm. The bacterial culture was re-inoculated in the well of 96-well flat-bottom plates containing fresh TSB supplemented with 3.0% NaCl and 0.5% glucose and incubated at 37 °C overnight without shaking. Four repeats were included for each strain. The biofilm was stained with crystal violet solution and measured as described in the Materials and Methods. The number of axial is the strain number of CFSa. *S. aureus* strain 15981 was used as a positive control of biofilm formation.

The formation of biofilms by *S. aureus* further complicates the treatment options for the MRSA infection on patients with cystic fibrosis. In our previous studies, we observed that different *S. aureus* isolates from patients with CF exhibited a distinct capacity of invading host cells [7] and led us to question whether these strains are able to form biofilms. To answer this question, we examined 50 different CF *S. aureus* isolates using clinical biofilm formation strain 15981 as a control [8]. At least 43 out of 50 CF *S. aureus* isolates exhibited a remarkable capacity of forming biofilm in vitro compared with the control (Figure 1). The strains CFSa8, 15, 18, 20, 36, and 20_2 strains showed a strong potency of biofilm formation with high reproducibility (Figure 1).

### 2.2. Random Identification of the cbiO Gene Required for Biofilm Formation by CA-MRSA on Plastic Surface In Vitro

**Figure 2:**
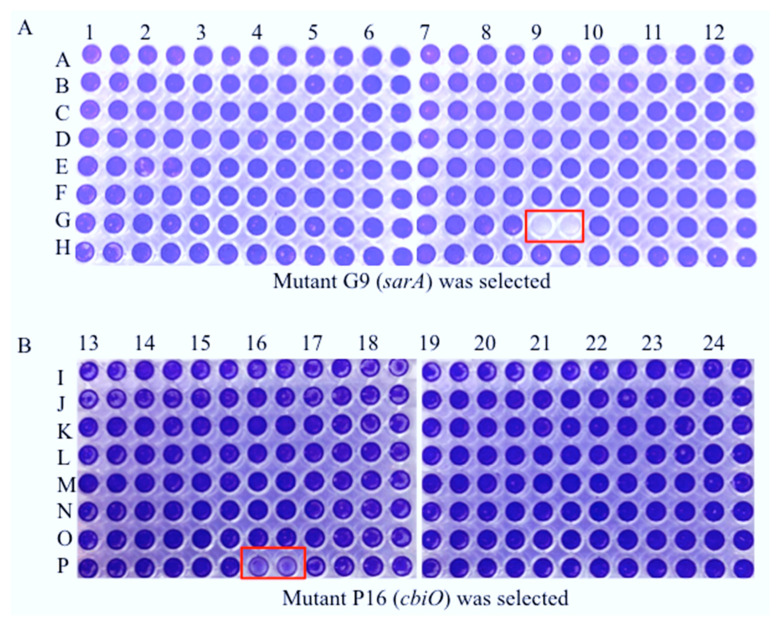
Screening Tn mutants with a weak capacity of biofilm formation using a Nebraska transposon mutant library. The Tn mutants were incubated in TSB supplemented with 3.0% NaCl, 0.5% glucose, and 5 μg/mL erythromycin at 37 °C overnight without shaking. The culture was re-inoculated in the well of 96-well flat-bottom plates containing fresh TSB supplemented with 3.0% NaCl, 0.5% glucose, and 5 μg/mL erythromycin, in duplicate, and incubated at 37 °C without shaking. The parental control JE2 was used as a positive control. The biofilm was stained with crystal violet solution and measured as described in the Materials and Methods. The red color box indicates a reduced biofilm formation by the mutant. (**A**) a mutant in G9 was identified and (**B**) a mutant in P16 was identified.

Though a few genes, such as those encoding fibronectin-binding-proteins FnBPA and FnBPB [9,10], a staphylococcal accessory regulator SarA [8,11], a teicoplanin-associated locus regulator (TcaR) and an intercellular adhesin locus regulator (IcaR) [12] have been reported as important for *S. aureus* biofilm formation, it remains vital to identify novel gene(s) that contribute to biofilm formation in order to better elucidate their molecular mechanisms. In this study, we screened a Nebraska transposon mutant library of CA-MRSA (JE2) composed of 1920 gene mutants for biofilm formation on a plastic surface in vitro using a 96-well format. Each mutant was duplicated during biofilm formation assays. Consistent with previous reports [13], we revealed that a Tn-mutation of *sarA* eliminated the capacity of JE2 to form a biofilm on the plastic surface (Figure 2A). Interestingly, we identified that a putative cobalt transporter ATP-binding subunit (*cbiO*) is critical for biofilm formation by the CA-MRSA JE2 strain (Figure 2B). The effect of Tn-mutation of *cbiO* on biofilm formation was further confirmed.

### 2.3. The Deletion Mutation of cbiO Dramatically Affected the Capacity of Biofilm Formation by an MRSA Strain, CFSa36, Isolated from a Pediatric Patient with Cystic Fibrosis

**Figure 3:**
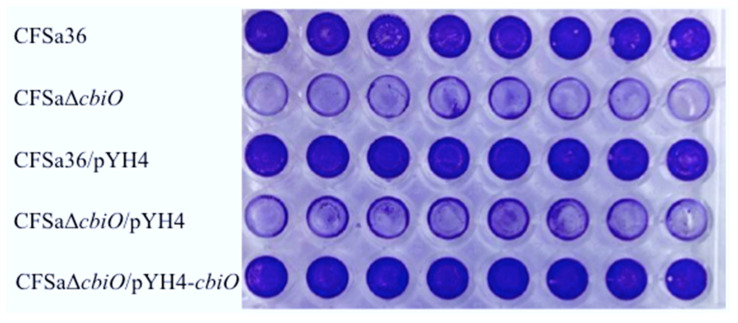
Effect of the *cbiO* deleted mutation and expression of *cbiO* *in trans* on biofilm formation by a clinical MRSA CFSa36. The *cbiO* null mutant CFSa36Δ*cbiO* and its parental control were incubated in TSB supplemented with 3.0% NaCl and 0.5% glucose CFSa36 at 37 °C overnight with shaking at 220 rpm. The *cbiO* null mutant CFSa36Δ*cbiO*/pYH4, complementary strain (CFSa36Δ*cbiO*/pYH4-*cbiO*), and their parental control CFSa36/pYH4 were incubated in TSB supplemented with 3.0% NaCl, 0.5% glucose, and 5 μg/mL erythromycin at 37 °C overnight with shaking at 220 rpm. The bacterial culture was re-inoculated in the well of 96-well flat-bottom plates containing fresh TSB supplemented with 3.0% NaCl and 0.5% glucose in the absence or presence of 5 μg/mL erythromycin and incubated at 37 °C overnight without shaking. Eight repeats were included for each strain. The biofilm was stained with crystal violet solution and measured as described in the Materials and Methods.

To pinpoint whether *cbiO* is important for biofilm formation by CFSa36, we first created a *cbiO* deletion mutant by using a homogenous recombination approach. Then, we performed biofilm formation assays after confirming a defined deletion mutation by using diagnostic PCR (Figure S1) and DNA sequencing of adjacent regions of *cbiO*. Indeed, the deletion of *cbiO* remarkably impaired the ability of CFSa36 strain to form biofilm (Figure 3).

### 2.4. The Introduction of cbiO Expression Plasmid Complemented the Biofilm Formation Capacity of the cbiO Null Mutant

To confirm that *cbiO* is necessary for biofilm formation and to eliminate the possibility that deleting the *cbiO* mutation had a polar effect, we performed a complementation study. The control strain carrying empty vector pYH4 (CFSa36/pYH4) had a similar capacity of forming biofilm to its parental control, CFSa36, whereas the *cbiO* null mutant carrying empty vector pYH4 significantly reduced the capacity of biofilm formation compared to the controls (Figure 3). However, the complementation of *cbiO* by introducing pYH4/*cbiO* into the *cbiO* null mutant fully restored the bacterial capacity of forming biofilm to the control level (Figure 3). Taken together, the above results demonstrated the role of *cbiO* in the biofilm formation by the MRSA CFSa36 strain.

### 2.5. The Addition of Extra Copper Ion (CuSO_4_) in a Chemically Defined Medium (CDM) Had No Influence on Bacterial Growth, but It Complemented the Biofilm Formation Capacity of the cbiO Knockout Mutant

**Figure 4:**
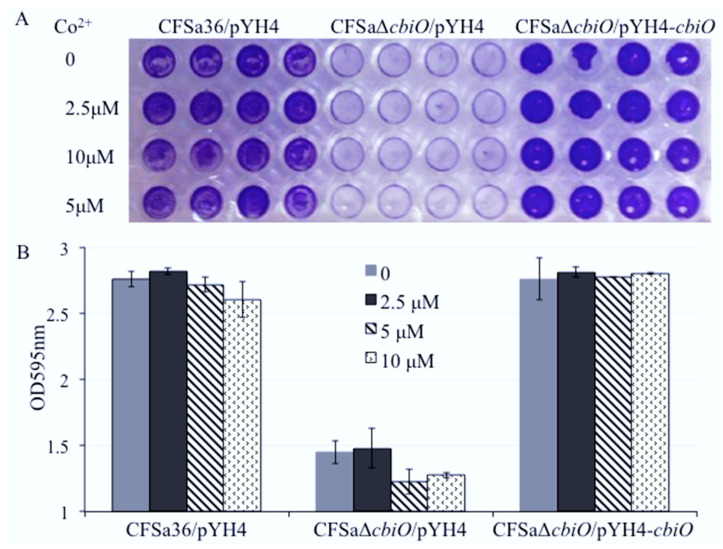
Impact of the addition of cobalt ions on the defect of biofilm formation by *cbiO* null mutant. The *cbiO* null mutant CFSa36Δ*cbiO*/pYH4, complementary strain (CFSa36Δ*cbiO*/pYH4-*cbiO*), and their parental control CFSa36/pYH4 were incubated in CDM supplemented with 5 μg/mL erythromycin at 37 °C overnight with shaking at 220 rpm. The cultures were re-inoculated in CDM with Erm^5^ and different concentrations of CoCl_2_, and incubated in a 96-well format at 37 °C overnight without shaking. Four repeats were included for each strain. (**A**) Impact of the addition of cobalt ions on the biofilm defect phenotype of *cbiO* null mutant, and (**B**) quantitatively determine the impact of the addition of cobalt ions on biofilm formation. The data were statistically analyzed with a T.TEST. The results are representative of at least three independent repeats.

The *cbiO* is a putative cobalt transporter ATP binding domain, thus, it is reasonable to elucidate whether the function of *cbiO* on biofilm formation is attributable to the addition of Co^2+^. We determined the effect of additional Co on biofilm formation by the *cbiO* knockout mutant. The addition of extra Co^2+^ ion in CDM had no impact on bacterial biofilm formation (Figure 4) and growth (Figure S2). This led us to speculate that other metal ions might be involved in the function of *cbiO* for biofilm formation. To test this possibility, we examined the impact that the addition of extra Cu^2+^, Ca^2+^, Mg^2+^, Zn^2+^, Ni^2+^, Mn^2+^, and Fe^3+^ ions in CDM had on *cbiO* knockout mutant biofilm formation.

**Figure 5:**
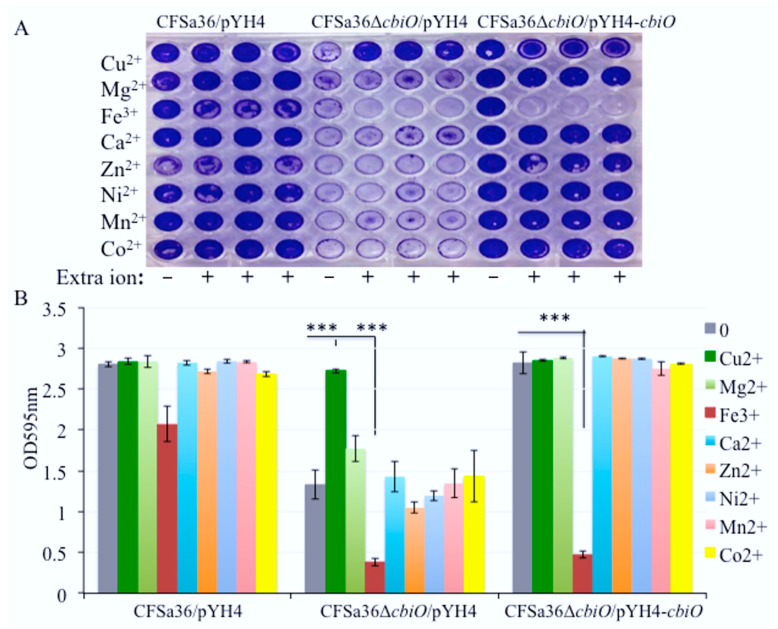
Impact of the addition of various metal ions on the defect of biofilm formation by *cbiO* null mutant. The *cbiO* null mutant CFSa36Δ*cbiO*/pYH4, complementary strain (CFSa36Δ*cbiO*/pYH4-*cbiO*), and their parental control CFSa36/pYH4 were incubated in CDM supplemented with 5 μg/mL erythromycin at 37 °C overnight with shaking at 220 rpm. The cultures were re-inoculated in CDM with Erm^5^ and different metal ions and incubated in a 96-well format at 37 °C overnight without shaking. Four repeats were included for each strain. (**A**) Impact of the addition of extra metal ions (2 μM Cu^2+^, 4.8 mM Mg^2+^, 200 μM Fe^3+^, 150 μM Cu^2+^, 18.8 μM Zn^2+^, 25 μM Ni^2+^, 18.8 μM Mn^2+^ or 5 μM Co^2+^) on the biofilm defect phenotype of *cbiO* null mutant, and (**B**) quantitatively determine the impact of the addition of different metal ions on the biofilm formation. The data were statistically analyzed with a T.TEST. The results are representative of at least three independent repeats. The symbol *** represents *p* < 0.001.

**Figure 6:**
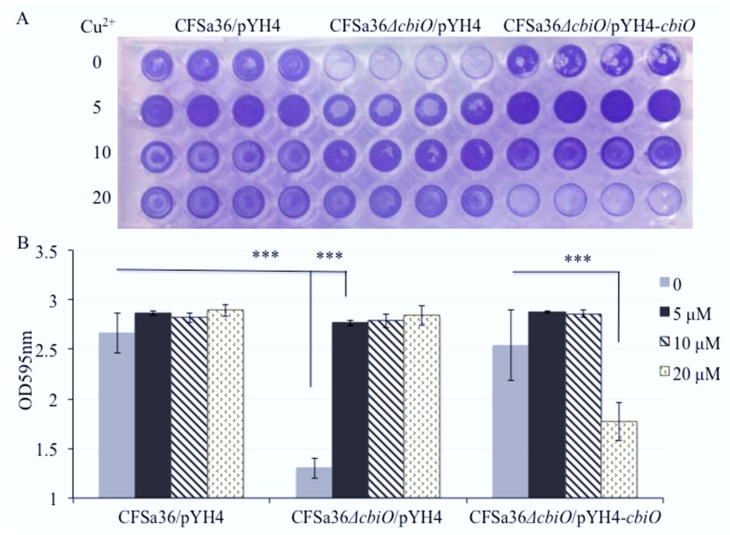
Effect of the addition of copper ions on the defect of biofilm formation by *cbiO* null mutant. The *cbiO* null mutant CFSa36Δ*cbiO*/pYH4, complementary strain (CFSa36Δ*cbiO*/pYH4-*cbiO*), and their parental control CFSa36/pYH4 were incubated in CDM supplemented with 5 μg/mL erythromycin at 37 °C overnight with shaking at 220 rpm. The cultures were re-inoculated in CDM with Erm^5^ and the different concentrations of CuSO_4_, and incubated in a 96-well format at 37 °C overnight without shaking. Four repeats were included for each strain. (**A**) Effect of the addition of extra copper ions on the biofilm defect phenotype of *cbiO* null mutant, and (**B**) quantitatively determine the effect of the addition of copper ions on biofilm formation. The data were statistically analyzed with a T.TEST. The results are representative of at least three independent repeats. The symbol *** represents *p* < 0.001.

Surprisingly, we found that the addition of extra Cu^2+^ ion in CDM totally restored the capacity of the *cbiO* knockout mutant to form a biofilm (Figure 5A,B). To further confirm the role of Cu^2+^ ions, we added different concentrations of Cu^2+^ ions in CDM and revealed that Cu^2+^ ions affected the biofilm formation of *cbiO* knockout mutant in a dose-dependent manner (Figure 6A,B).

**Figure 7:**
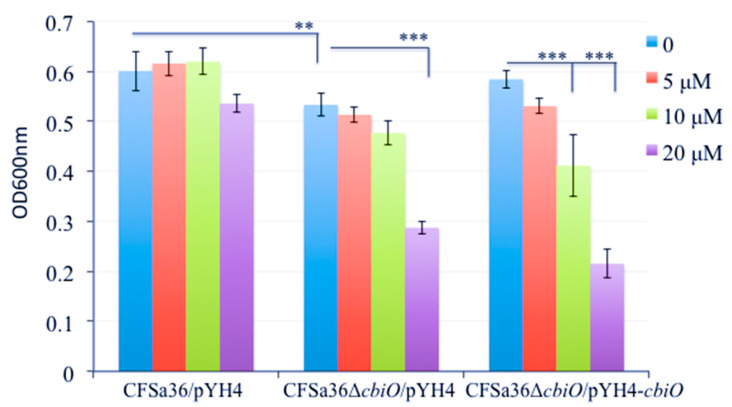
Effect of the addition of copper ions on the growth of *cbiO* null mutant, its complementary strain, and parental control. The *cbiO* null mutant CFSa36Δ*cbiO*/pYH4, complementary strain (CFSa36Δ*cbiO*/pYH4-*cbiO*), and their parental control CFSa36/pYH4 were incubated in CDM supplemented with 5 μg/mL erythromycin at 37 °C overnight with shaking at 220 rpm. The cultures were re-inoculated in CDM with Erm^5^ and the different concentrations of CuSO_4_, and incubated in a 96-well format at 37 °C overnight without shaking. Four repeats were included for each strain. The bacterial growth was determined by measuring the optical density of bacterial cultures at 600 nm. The data were statistically analyzed with a T.TEST. The results are representative of at least three independent repeats. The symbol ** represents *p* < 0.01 and the symbol *** represents *p* < 0.001.

To explore whether the defect of biofilm formation by the *cbiO* knockout mutant is a result of the growth defect, we examined the effect of *cbiO* on bacterial growth. The deletion mutation of *cbiO* dramatically inhibited the bacterial growth compared to the wild-type control (*p* = 0.00187), whereas the complementation of *cbiO* enhanced the bacterial growth compared to the *cbiO* null mutant (Figure 7). This led us to predict that the competition of *cbiO*’s role in biofilm formation by Cu^2+^ ions is attributed to its impact on growth. The amount of extra Cu^2+^ ions (5 to 20 µM) added in CDM exhibited no significant impact on the growth of wild-type control strain (Figure 7). Importantly, the addition of extra 5 to 10 µM Cu^2+^ ions in CDM had no influence on the growth defect of the *cbiO* knockout mutant, and 20 µM copper ions significantly inhibited growth (Figure 7). Interestingly, the addition of extra 10 to 20 µM Cu^2+^ ions also remarkably inhibited the growth of the *cbiO* complementary strain (Figure 7). The above data indicate that the Cu^2+^ ions could specifically complement the function of *cbiO* in biofilm formation.

### 2.6. The Addition of Extra Fe^3+^ in CDM Enhanced the Effect of cbiO Null Mutation on Biofilm Formation

**Figure 8:**
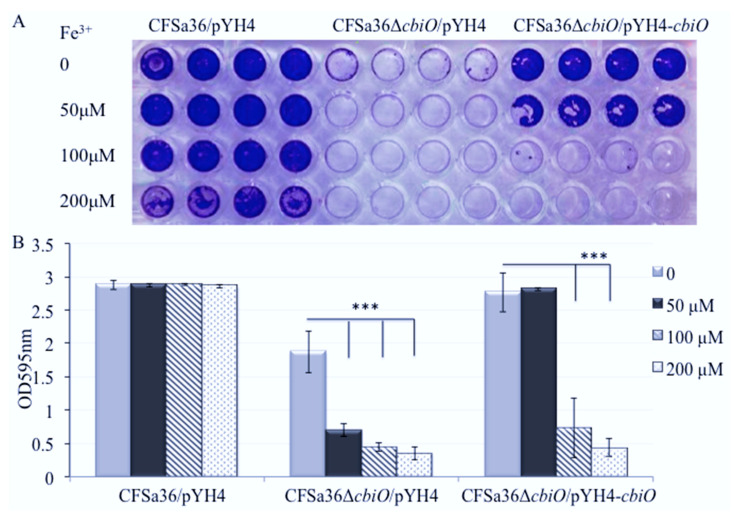
Effect of the addition of iron ions on the defect of biofilm formation by *cbiO* null mutant. The *cbiO* null mutant CFSa36Δ*cbiO*/pYH4, complementary strain (CFSa36Δ*cbiO*/pYH4-*cbiO*), and their parental control CFSa36/pYH4 were incubated in CDM supplemented with 5 μg/mL erythromycin at 37 °C overnight with shaking at 220 rpm. The cultures were re-inoculated in CDM with Erm^5^ and the different concentrations of FeCl_3_, and incubated in a 96-well format at 37 °C overnight without shaking. Four repeats were included for each strain. (**A**) Effect of the addition of iron ions on the biofilm defect phenotype of *cbiO* null mutant, and (**B**) quantitatively determine the effect of the addition of iron ions on biofilm formation. The data were statistically analyzed with a T.TEST. The results are representative of at least three independent repeats. The symbol *** represents *p* < 0.001.

Furthermore, our results showed that the addition of extra Fe^3+^ in CDM completely eliminated biofilm formation by the *cbiO* null mutant (Figure 8A,B). The inhibition of biofilm formation of the *cbiO* null mutant by Fe^3+^ exhibited dose dependence (Figure 8A,B). The addition of extra 100 or 200 µM of Fe^3+^ also alleviated the biofilm formation of the *cbiO* complementary strain (Figure 8A,B). However, the addition of extra 200µM Fe^3+^ had no influence on bacterial growth (Figure S4).

## 3. Discussion

It is well established that *S. aureus* is the most common bacterial pathogen associated with the respiratory secretions of patients with CF [14,15,16], particularly in pediatric patients with CF [17]. The prevalence of *S. aureus* in patients with CF has increased in Europe and in the US, which may be attributed to the continued emergence of antibiotic-resistant *S. aureus* isolates, particularly methicillin-resistant *S. aureus* (MRSA) [18,19]. Despite administraton of antibiotics with demonstrated therapeutic minimal inhibitory conconcentrations in vitro, *S. aureus* isolates elicit persistent colonization in the patients with CF an effect which may be attributable to biofilm formation [20]. Moreover, the increased use of medical implants in health care is strongly associated with elevated biofilm-related persistent infections [21,22]. The biofilm formation by *S. aureus* is controlled by different regulators such as SarA [8,11], TacR, and IcaR [12], and affected by various environmental factors including metal ions [6].

In this study, we found that most *S. aureus* isolates from pediatric patients with CF possess a strong capacity of generating biofilm on plastic surfaces, suggesting that this characteristic of CF *S. aureus* isolates might contribute to the propensity of persistent bacterial colonization in patients with CF. This finding also highlights the importance of disinfecting reusable oral plastic devices used in CF care procedures.

More importantly, we are the first to demonstrate that a putative cobalt transporter ATP-binding domain (*cbiO*) plays an important role in biofilm formation for CFSa36 strain using genetic loss-of-function and gain-of-function strategies. Interestingly, our results indicated that the contribution of *cbiO* to biofilm formation is attributable to its modulation of copper ions and is not due to cobalt ions in our experimental environment. These findings should provide a novel target and/or an alternative strategy to combat biofilm formation by *S. aureus*, especially MRSA in pediatric patients with CF. Further studies are required to determine whether *cbiO* is a dominant determinant for persistent MRSA colonization in patients with CF.

Although *cbiO* is a putative cobalt transporter ATP-binding domain, the addition of extra cobalt ions in the chemical defined medium had no influence on defective biofilm formation by *cbiO* knockout mutant. One explanation is that the null mutation of *cbiO* may eliminate the bacterial cell’s ability to transport cobalt ions. However, we belive that *cbiO* transportation of cobalt ions is not involved in biofilm formation, as the addition of cobalt ions in CDM had no impact on biofilm formation in either *cbiO* null mutant or in the parental control. Surprisingly, we found the addition of an extra 5–20 µM CuSO_4_ in CDM did not affect biofilm formation of the wild type control. In contrast, the copper ions were able to entirely complement the capacity of biofilm formation by the *cbiO* null mutant in a dose-dependent manner. It was observed that the *cbiO* null mutation inhibited bacterial growth, suggesting that the influence of *cbiO* on biofilm formation may result from the growth defect of the *cbiO* knockout mutant. However, the copper ions enabled the *cbiO* knockout mutant to restore the capacity of biofilm formation, whereas the addition of the same amount of CuSO_4_ had no obvious impact on bacterial growth. Taken together, these results indicated the specific role of copper ions in *cbiO*-mediated *S. aureus* biofilm formation. We postulate that the copper ions possibly function as a cofactor to mediate *S. aureus* biofilm formation. Our results also showed that the addition of extra 10 to 20 µM CuSO_4_ in CDM dramatically inhibited the growth of *cbiO* complementary strain. One possible explanation is that the overexpression of *cbiO* in trans could lead to a higher amount of copper ion within the bacterial cells, which results in a toxic effect on bacterial cells. In future studies, we will determine how *cbiO* affects the function of copper ions, whether *cbiO* directly binds to copper, and how copper ions complement the function of *cbiO* in biofilm formation.

Metal ions have been explored for their application in antibacterial activity and elimination of biofilms in different bacterial systems [23,24,25,26,27]. The role of metal ions on *S. aureus* biofilm formation has been reviewed elsewhere [6]. In this study, we revealed that the addition of additional 100 to 300 µM FeCl_3_ had no remarkable effect on bacterial growth, but did significantly decrease the ability of *S. aureus* CFSa36 to form biofilms in a dose-dependent manner (Figure 8). However, this result is inconsistent with the previous findings that iron chloride (FeCl_3_) enhanced the biofilm formation by *S. aureus* Xen 31 strain [28], which is possibly due to the different genetic background between CFSa36 and Xen 31. Recently, the Leileg lab has reported that metal ions, including copper and zinc, could alleviate the hydrophobicity of *Bacillus subtilis* to affect the stability of its biofilms [29]. However, in this study, we found that with the exception of FeCl_3_, the addition of extra 18.8 µM ZnCl_2_, 4.8 mM MgCl2, 150 µM CaCl_2_, 25 µM NiSO_4_, and 18.8 µM MnCl_2_ in CDM had no impact on *S. aureus* biofilm formation. The inconsistent findings are possibly due to different bacterial species, genetic backgrounds, and experimental conditions such as culture medium and time. It has been reported that the Newman strain of *S. aureus* exhibited a limited ability to form biofilms when cultured in the presence of calcium chelators, whereas *S. aureus* strain 10883 generated robust biofilms in the same conditions [30]. In addition, we cannot exclude the possibility that these ions affect the stability of mature *S. aureus* biofilms.

In this study we screened a Nebraska Transposon mutation library to identify any mutants with lost or weak biofilm formation on a plastic surface in vitro. Consistent with a previous report [31], we identified that SarA is necessary for biofilm formation by USA300 CA-MRSA JE2 strain (Figure 2). This result reassured us that the random identification of *cbiO* involved in biofilm formation is worthy of further investigation. Our results are different from the Kaplan laboratory’s report of identifying genes that are involved in biofilm formation impacted by low-dose amoxicillin using the same Tn-mutant library [13]. This is likely due to the discrepancy of experimental conditions such as bacterial culture conditions and the surface of biofilm formation. Although our results clearly indicated the requirement of *cbiO* for the biofilm formation by CFSa36 strain (a human clinical MRSA) and probable JE2 strain (CA-MRSA), we cannot exclude the possibility that *cbiO* is indispensable for other *S. aureus* strains, including MRSA, to generate biofilm. We are in the process of investigating whether the role of *cbiO* in biofilm formation is affected by the genetic backgrounds of *S. aureus* isolates.

Our study adds to the growing reports urging the development of biofilm inhibitors. Promising agents include zinc oxide nanoparticles, proteinase K and hamamelitannin. These agents are reported to transcriptionally modulate biofilm and quorum sensing genes [32]. Others have found that 1, 2, and 4-oxadiazole derivatives have potent MRSA biofilm inhibition, perhaps due to inhibition of the transpeptidase sortase SrtA [33]. Our study is unique in the identification of a hypothetical cobalt transporter that regulates biofilm formation in a coppoer ion dependent manner.

In conclusion, we demonstrated that most *S. aureus* isolates from pediatric patients with cystic fibrosis possess a potent capacity to generate biofilms in vitro. We are the first to reveal that a cobalt transporter ATP-binding domain (*cbiO*) plays a critical role in biofilm formation of the MRSA CFSa36 strain, and the addition of copper ions could complement the function of *cbiO* for biofilm formation in a dose-dependent manner. In contrast, iron chloride had a detrimental effect on biofilm formation by the *cbiO* null mutant. These findings suggest that *cbiO* may be a potential target for combating *S. aureus*, including MRSA biofilm-associated infections, and may be used to prevent *S. aureus* colonization in pediatric patients with CF. Strategies, such as chelation, to limit copper ion availability in the airway milieu might impair *S. aureus* biofilm formation.

## 4. Materials and Methods

### 4.1. Bacterial Strains, Plasmids and Growth Media

The bacterial strains and plasmids used in this study are listed in Table 1. CFSa36 is a clinical isolate of MRSA with an underterminded sequencing type (ST) [7]. *Escherichia coli* DC10B (gift of T.J. Foster) served as the host for all in vitro recombinant DNA [34]. *E. coli* transformants were selected on Brain Heart Infusion (BHI; Difco) agar containing erythromycin (100 µg/mL) or Luria-Bertani agar containing ampicillin (100 µg/mL). *S. aureus* was cultured in Trypticase Soy Broth (TSB; Difco) or on TSA agar at 37 °C with appropriate antibiotics. All bacterial cell cultures were incubated with shaking at 220 RPM. *S. aureus* transformants were selected on TSA containing chloramphenicol (10 µg/mL) or erythromycin (5 µg/mL).

TSB supplemented with 3.0% NaCl and 0.5% glucose was used for static biofilm formation studies. To determine the impact of metal ions on biofilm formation, a chemically defined medium (CDM) was utilized for cultivation of *S. aureus*. All amino acids in CDM were L-amino acids. Based upon the concentration of these ions in CDM, where noted, extra CoCl_2_ (2.5–10 µM), CuSO_4_ (2–20 µM), MgCl_2_ (4.8 mM), CaCl_2_ (150 µM), ZnCl_2_ (18.8 µM), MnCl_2_ (18.8 µM), NiSO_4_ (25 µM), or FeCl_3_ (50–200 µM) was added to CDM to determine their impact on bacterial growth and biofilm fomation.

### 4.2. Construction of the cbiO Gene Deletion Mutant and the cbiO Gene Complemented Strains

Deletion of *cbiO* was carried out following the pKOR1 allelic exchange protocol as described [36] and primers sets cbiO-pKOR1 L–For/Rev listed in Table 1. The R-For primer was synthesized with a 5′ phosphate group. Each PCR fragment was purified, and the two fragments were ligated together with T4 DNA ligase (Promega, Madison, WI, USA). The ligation product was mixed with BP Clonase, per the manufacturer’s instructions, and plasmid pKOR1, incubated at 25 °C overnight, then transformed into *E. coli* DC10B. The pKOR1-*cbiO*KO plasmid was subsequently transformed into *S. aureus* CFSa36. Large colonies were re-streaked to fresh TSA plates and deletion of *cbiO* was confirmed through diagnostic PCR (Figure S1) and DNA sequencing.

In order to examine whether the expression of *cbiO* in trans can complement the effect of the mutation of the respective endogenous gene, we constructed recombinant plasmids, pYH4/*cbiO* by cloning the *cbiO* coding region (which was obtained by PCR) into pYH4 under the control of *xyl*/*tet* promoter [37], and electroporated it into the *cbiO* knockout mutant, resulting in CFSa36/pYH4-*cbiO* complementation strain in Table 1. The recombinant plasmid DNA were isolated from the complementary strain and confirmed by PCR and DNA sequencing.

To determine the impact of *cbiO* and metal ions on growth, the bacterial growth was monitored at 37 °C by measuring the optical density at 600 nm with a SpectraMax Plus spectrophotometer as described [38].

### 4.3. Static Biofilm Formation Assays

The biofilm formation assays were conducted in a 96-well format under static conditions as described [39]. Briefly, the wells of 96-well flat-bottom plates (Sarstedt Inc., Newton, NC, USA) were coated with 5% human plasma in carbonate–bicarbonate buffer (pH 9.6). Each Tn-mutant from a Nebraska transposon mutant library stock plate was inoculated 1:50 in each well containing 200 µL of TSB supplemented with 3.0% NaCl and 0.5% glucose or CDM with a supplementation of erythromycin (5 µg/mL) accordingly and incubated at 37 °C without shaking for 24 h. Each mutant was re-inoculated 1:200 in duplicate in the coated well containing the same culture medium and conditions for 24 h. The bacterial growth was determined by measuring the optical density at 600 nm with a SpectraMax Plus spectrophotometer. Then, the planktonic bacterial solution was carefully removed from each well and washed three times with PBS. The biofilm biomass in each well was fixed with 200 µL of 100% ethanol and dried, stained for 2 min with 200 µL of Gram’s crystal violet solution, then gently rinsed three times with PBS, air dried, and images were taken to record the results. The bound biofilm biomass in each well was dissolved with 100 µL of 100% ethanol and quantitively measured at 595 nm with a SpectraMax Plus spectrophotometer.

To ensure the reproducibility, each mutant was tested in duplicate during original screening of the transposon mutant library and repeated at least three times and each included at least triplites for further studies.

### 4.4. Data Analysis

The independent samples of biofilms were statistically analyzed using a T.TEST with an alpha level <0.001 considered significant. For data figures with more than two independent samples, a one-way ANOVA analysis with a post-hoc Tukey HSD test was used to determine if there was statistical significance between samples, with an alpha level ≤0.001 considered significant.

## Figures and Tables

**Table 1:** Bacterial strains, plasmids, and primers used in this study.

Strain, Plasmid or Primer	Relevant Characteristics	Reference
*Strain* DC10B	Dam-*E. coli*	[34]
Nebraska Tn-mutant library	1920 genes transposon mutants, Erm^R^	[35]
JE2	USA300 CA-MRSA isolate	[35]
15983	Clinical *S. aureus* biofilm formation strain	[8]
CFSa isolates (49) CFSa36	Human clinical MRSA isolate Human clinical MRSA isolate, *sigB^+^*/*rsbU*^+^	[7] [7]
CFSa36Δ*cbiO*	CFSa36 *cbiO* deletion mutant	This study
CFSa36Δ*cbiO*/pYH4	CFSa36 *cbiO* deletion mutant with pYH4; Erm^R^	This study
CFSa36Δ*cbiO*/pYH4-*cbiO*	CFSa36 *cbiO* deletion mutant with pYH4-*cbiO*; Erm^R^	This study
*Plasmid*		
pKOR1	Temperature sensitive inducible allelic exchange plasmid for *S. aureus*; Cm^R^	[36]
pKOR1-*cbiO*	pKOR1 with in-frame *cbiO* upstream/downstream deletion region; Cm^R^	This study
pYH4	Shuttle vector with Tc inducible promoter; Erm^R^	[37]
pYH4-*cbiO*	*cbiO* cloned downstream of pYH4 *tet* promoter; Erm^R^	This study
*Primer*		
cbiO_pKOR1_L_F	5′GGGGACAAGTTTGTACAAAAAAGCAGGCTGTGCAAACAC CCAAAGATATG3′	
cbiO_pKOR1_R_R	5′GGGGACCACTTTGTACAAGAAAGCTGGGTGCTGACATGA TGAAAGTGCG3′	
cbiO_L_R	5′GAAGGGCTGGTGGATCAAC3′	
cbiO_R_F	5′Phos/CACTTGTCTCTCTCCTTTAC3′	
cbiO_F	5′AGCTTTGTTTAAACGTGGAGGATAAGAATTCAG5′	
cbiO_R	5′AGGCGCGCCTCATAGTTGATCCACCAG CC3′	

Erm^R^: means resistance to erythromycin.

## Supplementary Materials

The following are available online at https://www.mdpi.com/article/10.3390/pathogens10111363/s1, Figure S1. Determination of *cbiO* deletion mutation by using diagnostic PCR. The genomic DNAs were isolated from both the wild-type control and the mutant strain and utilized as templates. The cbiO gene-specific primers (Lane 1–3: cbioFor/cbioRev primers), and both upstream and downstream of *cbiO* gene-specific primers (Lane 4–6: cibo-pKOR1_LF/ cbio-pKOR1_RR primers) were used for PCR. Lane 1 and 4: CFSa36; lane 2 and 5: CFSa36ΔcbiO; lane 3 and 6: negative controls. M: 1 kb DNA ladder; Figure S2. Impact of the addition of cobalt ions on the growth of *cbiO* null mutant, its complementary strain, and parental control. The *cbiO* null mutant CFSa36 *cbiO*/pYH4, complementary strain (CFSa36 *cbiO*/pYH4-cbiO), and their parental control CFSa36/pYH4 were incubated in CDM supplemented with 5 μg/mL erythromycin at 37 °C overnight with shaking at 220 rpm. The cultures were re-inoculated in CDM with Erm^5^ and the different concentrations of CoCl_2_, and incubated in a 96-well format at 37 °C overnight without shaking. Four repeats were included for each strain. The data were statistically analyzed with a T.TEST. The bacterial growth was determined by measuring the optical density of bacterial cultures at 600 nm. The results are representative of at least three independent repeats. Figure S3. Impact of the addition of metal ions on the growth of *cbiO* null mutant, its complementary strain, and parental control. The *cbiO* null mutant CFSa36 *cbiO*/pYH4, complementary strain (CFSa36 *cbiO*/pYH4-cbiO), and their parental control CFSa36/pYH4 were incubated in CDM supplemented with 5 μg/mL erythromycin at 37 °C overnight with shaking at 220 rpm. The cultures were re-inoculated in CDM with Erm^5^ and the different concentrations of metal ions (2 μM Cu^2+^, 4.8 mM Mg^2+^, 200 μM Fe^3+^, 150 μM Cu^2+^, 18.8 μM Zn^2+^, 25 μM Ni^2+^, 18.8 μM Mn^2+^ or 5 μM Co^2+^) incubated in a 96-well format at 37 °C overnight without shaking. Four repeats were included for each strain. The bacterial growth was determined by measuring the optical density of bacterial cultures at 600 nm. The data were statistically analyzed with a T.TEST. The results are representative of at least three independent repeats. Figure S4. Impact of the addition of iron ions on the growth of *cbiO* null mutant, its complementary strain, and parental control. The *cbiO* null mutant CFSa36 *cbiO*/pYH4, complementary strain (CFSa36 *cbiO*/pYH4-cbiO), and their parental control CFSa36/pYH4 were incubated in CDM supplemented with 5 μg/mL erythromycin at 37 °C overnight with shaking at 220 rpm. The cultures were re-inoculated in CDM with Erm^5^ and the different concentrations of FeCl_3_, and incubated in a 96-well format at 37 °C overnight without shaking. Four repeats were included for each strain. The bacterial growth was determined by measuring the optical density of bacterial cultures at 600 nm. The data were statistically analyzed with a T.TEST. The results are representative of at least three independent repeats.

## References

[1] Li C., Wu Y., Riehle A., Ma J., Kamler M., Gulbins E., Grassmé H. (2017). Staphylococcus aureus Survives in Cystic Fibrosis Macro-phages, Forming a Reservoir for Chronic Pneumonia. Infect. Immun..

[2] Kahl B.C. (2010). Impact of Staphylococcus aureus on the pathogenesis of chronic cystic fibrosis lung disease. Int. J. Med. Microbiol..

[3] Cascioferro S., Carbone D., Parrino B., Pecoraro C., Giovannetti E., Cirrincione G., Diana P. (2021). Therapeutic Strategies to Counteract Antibi-otic Resistance in MRSA Biofilm-Associated Infections. ChemMedChem.

[4] Dasenbrook E.C., Merlo C.A., Diener-West M., Lechtzin N., Boyle M.P. (2008). Persistent methicillin-resistant Staphylococcus aure-us and rate of FEV1 decline in cystic fibrosis. Am. J. Respir. Crit. Care Med..

[5] Boles B.R., Horswill A.R. (2011). Staphylococcal biofilm disassembly. Trends Microbiol..

[6] Liu Y., Zhang J., Ji Y. (2020). Environmental factors modulate biofilm formation by Staphylococcus aureus. Sci. Prog..

[7] Liu Y., Zhang J., Zhong D., Ji L., Yang J., Phillips J., Ji Y. (2016). Characterization of Staphylococcus aureus isolates from pediatric patients with cystic fibrosis. World J. Microbiol. Biotechnol..

[8] Valle J., Toledo-Arana A., Berasain C., Ghigo J.-M., Amorena B., Penadés J.R., Lasa I. (2003). SarA and not σB is essential for biofilm development by Staphylococcus aureus. Mol. Microbiol..

[9] O’Neill E., Humphreys H., O’Gara J.P. (2009). Carriage of both the fnbA and fnbB genes and growth at 37 degrees C promote FnBP-mediated biofilm development in meticillin-resistant Staphylococcus aureus clinical isolates. J. Med. Microbiol..

[10] O’Neill E., Pozzi C., Houston P., Humphreys H., Robinson D.A., Loughman A., Foster T.J., O’Gara J.P. (2008). A novel Staphylococ-cus aureus biofilm phenotype mediated by the fibronectin-binding proteins, FnBPA and FnBPB. J. Bacteriol..

[11] Beenken K.E., Blevins J.S., Smeltzer M.S. (2003). Mutation of sarA in Staphylococcus aureus Limits Biofilm Formation. Infect. Immun..

[12] Jefferson K.K., Pier D.B., Goldmann D.A., Pier G.B. (2004). The Teicoplanin-Associated Locus Regulator (TcaR) and the Intercellular Adhesin Locus Regulator (IcaR) Are Transcriptional Inhibitors of the ica Locus in Staphylococcus aureus. J. Bacteriol..

[13] Mlynek K.D., Callahan M.T., Shimkevitch A.V., Farmer J.T., Endres J.L., Marchand M., Bayles K.W., Horswill A.R., Kaplan J.B. (2016). Effects of Low-Dose Amoxicillin on Staphylococcus aureus USA300 Biofilms. Antimicrob. Agents Chemother..

[14] Lyczak J.B., Cannon C.L., Pier G.B. (2002). Lung Infections Associated with Cystic Fibrosis. Clin. Microbiol. Rev..

[15] LiPuma J.J. (2010). The Changing Microbial Epidemiology in Cystic Fibrosis. Clin. Microbiol. Rev..

[16] Phillips J.R., Tripp T.J., Regelmann W.E., Schlievert P.M., Wangensteen O.D. (2006). Staphylococcal alpha-toxin causes increased tracheal epithelial permeability. Pediatr. Pulmonol..

[17] Andersen D.H. (1949). The Present Diagnosis and Therapy of Cystic Fibrosis of the Pancreas. Proc. R. Soc. Med..

[18] Yagci S., Hascelik G., Dogru D., Ozcelik U., Sener B. (2013). Prevalence and genetic diversity of Staphylococcus aureus small-colony variants in cystic fibrosis patients. Clin. Microbiol. Infect..

[19] Emerson J., McNamara S., Buccat A.M., Worrell K., Burns J.L. (2010). Changes in cystic fibrosis sputum microbiology in the United States between 1995 and 2008. Pediatr. Pulmonol..

[20] Vu-Thien H., Hormigos K., Corbineau G., Fauroux B., Corvol H., Moissenet D., Vergnaud G., Pourcel C. (2010). Longitudinal survey of Staphylococcus aureus in cystic fibrosis patients using a multiple-locus variable-number of tandem-repeats analysis method. BMC Microbiol..

[21] Costerton J.W., Stewart P., Greenberg E. (1999). Bacterial Biofilms: A Common Cause of Persistent Infections. Science.

[22] O’Grady N.P., Alexander M., Dellinger E.P., Gerberding J.L., Heard S.O., Maki D.G., Masur H., McCormick R.D., Mermel L.A., Pearson M.L. (2002). Guidelines for the prevention of intravascular catheter-related infections. Centers for Disease Control and Prevention. MMWR Recomm. Rep..

[23] Lemire J.A., Harrison J., Turner R.J. (2013). Antimicrobial activity of metals: Mechanisms, molecular targets and applications. Nat. Rev. Genet..

[24] Dinh Т.L., Akhmetova G.R., Martykanova D.S., Rudakova N.L., Sharipova М.R. (2019). Influence of Divalent Metal Ions on Biofilm Formation by Bacillus subtilis. BioNanoScience.

[25] Sirelkhatim A., Mahmud S., Seeni A., Kaus N.H.M., Ann L.C., Bakhori S.K.M., Hasan H., Mohamad D. (2015). Review on Zinc Oxide Nanoparticles: Antibacterial Activity and Toxicity Mechanism. Nano-Micro Lett..

[26] Dupont C.L., Grass G., Rensing C. (2011). Copper toxicity and the origin of bacterial resistance—new insights and applications. Metallomics.

[27] Hsueh Y.-H., Ke W.-J., Hsieh C.-T., Lin K.-S., Tzou D.-Y., Chiang C.-L. (2015). ZnO Nanoparticles Affect Bacillus subtilis Cell Growth and Biofilm Formation. PLoS ONE.

[28] Ahire J.J., Dicks L.M.T. (2014). Nisin Incorporated With 2,3-Dihydroxybenzoic Acid in Nanofibers Inhibits Biofilm Formation by a Methicillin-Resistant Strain of Staphylococcus aureus. Probiotics Antimicrob. Proteins.

[29] García C.F., Kretschmer M., Lozano-Andrade C.N., Schönleitner M., Dragos A., Kovács Á.T., Lieleg O. (2020). Metal ions weaken the hydrophobicity and antibiotic resistance of Bacillus subtilis NCIB 3610 biofilms. NPJ Biofilms Microbiomes.

[30] Abraham N.M., Lamlertthon S., Fowler V.G., Jefferson K.K. (2012). Chelating agents exert distinct effects on biofilm formation in Staphylococcus aureus depending on strain background: Role for clumping factor B. J. Med. Microbiol..

[31] Zielinska A.K., Beenken K.E., Mrak L.N., Spencer H.J., Post G.R., Skinner R.A., Tackett A.J., Horswill A.R., Smeltzer M.S. (2012). sarA-mediated repression of protease production plays a key role in the pathogenesis of Staphylococcus aureus USA300 isolates. Mol. Microbiol..

[32] Abd El-Hamid M.I., YEl-Naenaeey E.S., MKandeel T., Hegazy W.A.H., Mosbah R.A., Nassar M.S., Bakhrebah M.A., Abdulaal W.H., Alhakamy N.A., Bendary M.M. (2020). Promising Antibiofilm Agents: Recent Breakthrough against Biofilm Producing Methi-cillin-Resistant Staphylococcus aureus. Antibiotics.

[33] Carbone D., Parrino B., Cascioferro S., Pecoraro C., Giovannetti E., Di Sarno V., Musella S., Auriemma G., Cirrincione G., Diana P. (2021). 1,2,4-Oxadiazole Topsentin Analogs with Antiproliferative Activity against Pancreatic Cancer Cells, Targeting GSK3β Kinase. ChemMedChem.

[34] Monk I.R., Shah I.M., Xu M., Tan M.-W., Foster T.J. (2012). Transforming the Untransformable: Application of Direct Transformation to Manipulate Genetically Staphylococcus aureus and Staphylococcus epidermidis. mBio.

[35] Fey P.D., Endres J.L., Yajjala V.K., Widhelm T.J., Boissy R.J., Bose J.L., Bayles K.W. (2013). A genetic resource for rapid and compre-hensive phenotype screening of nonessential Staphylococcus aureus genes. mBio.

[36] Bae T., Schneewind O. (2006). Allelic replacement in Staphylococcus aureus with inducible counter-selection. Plasmid.

[37] Huang J., O’Toole P., Shen W., Amrine-Madsen H., Jiang X., Lobo N., Palmer L.M., Voelker L., Fan F., Gwynn M.N. (2004). Novel Chromosomally Encoded Multidrug Efflux Transporter MdeA in Staphylococcus aureus. Antimicrob. Agents Chemother..

[38] Yan M., Yu C., Yang J., Ji Y. (2011). The essential two-component system YhcSR is involved in regulation of the nitrate respira-tory pathway of Staphylococcus aureus. J. Bacteriol..

[39] Cassat J.E., Smeltzer M.S., Lee C.Y. (2014). Investigation of Biofilm Formation in Clinical Isolates of Staphylococcus aureus. Methods Mol. Biol..

