# Supplementary material for: Identification of cbiO Gene Critical for Biofilm Formation by MRSA CFSa36 Strain Isolated from Pediatric Patient with Cystic Fibrosis
Source: Pathogens. 2021 Oct 21;10(11):1363. doi: 10.3390/pathogens10111363 (PMC8622116; doi:10.3390/pathogens10111363)
Supplement: Supplementary file 1 [file pathogens-10-01363-s001.zip › pathogens-1427478-supplementary.pdf]

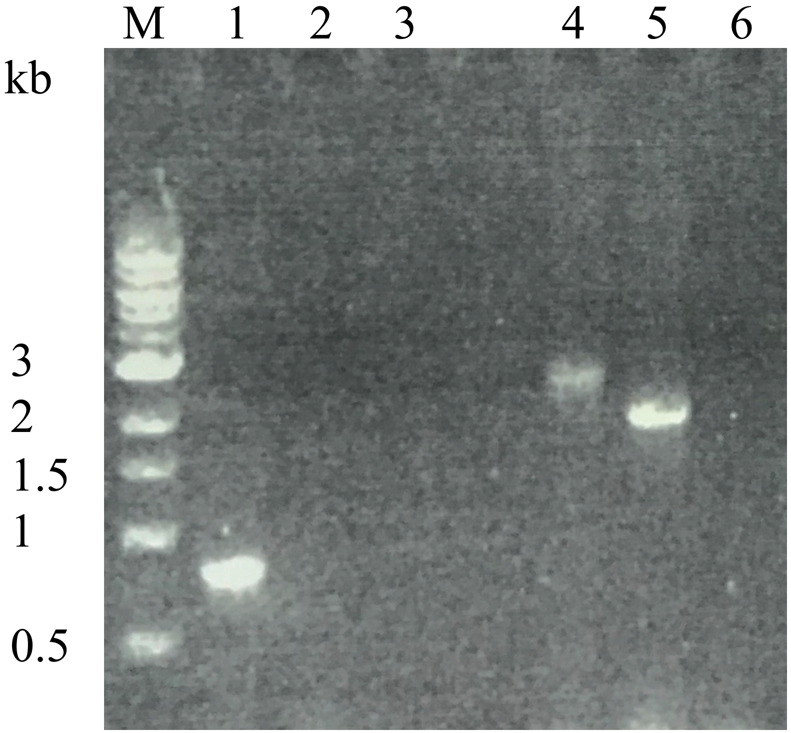

Figure S1

Lane 1 & 4: CFSa36; lane 2 & 5: CFSa36ΔcbiO; lane 3 & 6: negative controls.
M: 1 kb DNA ladder. Lane 1-3: cibo-pKOR1_LF/ cbio-pKOR1_RR primers
Lane 4 – 6: cbioFor/cbioRev primers.

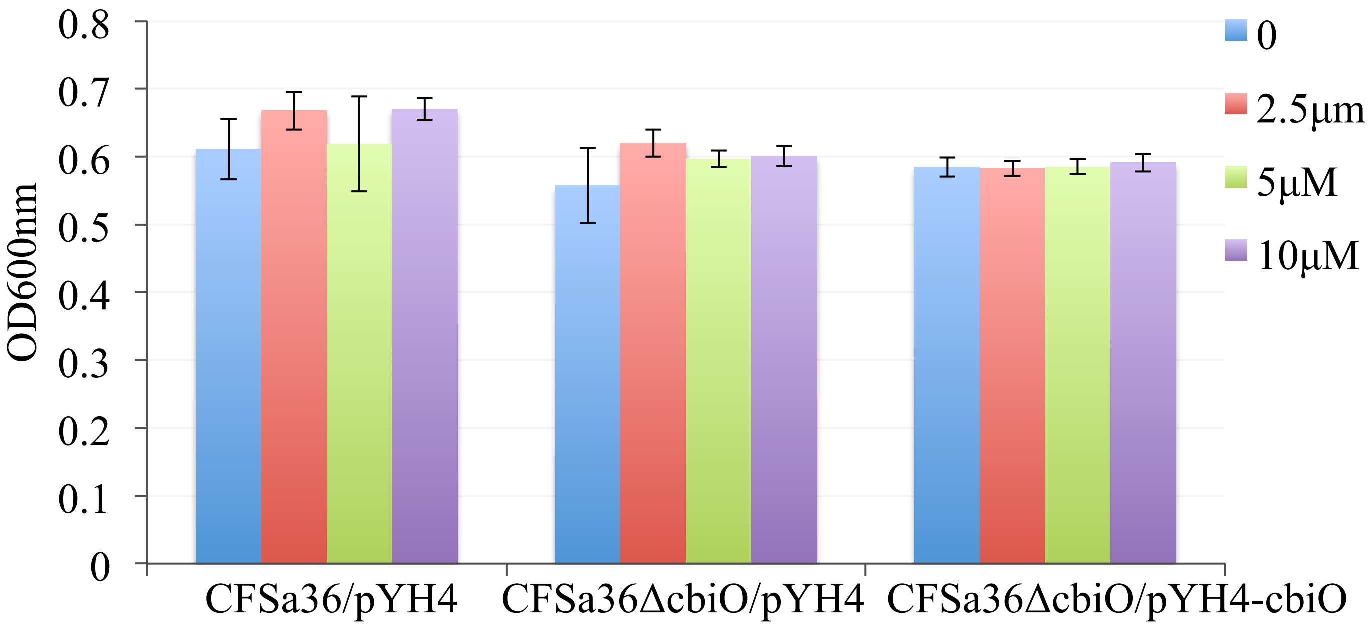
0.8
0.7
0.6
0.5
0.4
0.3
0.2
0.1
0
OD600nm
CFSa36/pYH4
CFSa36ΔcbiO/pYH4
CFSa36ΔcbiO/pYH4-cbiO
0
2.5µm
5µM
10µM

Figure S2

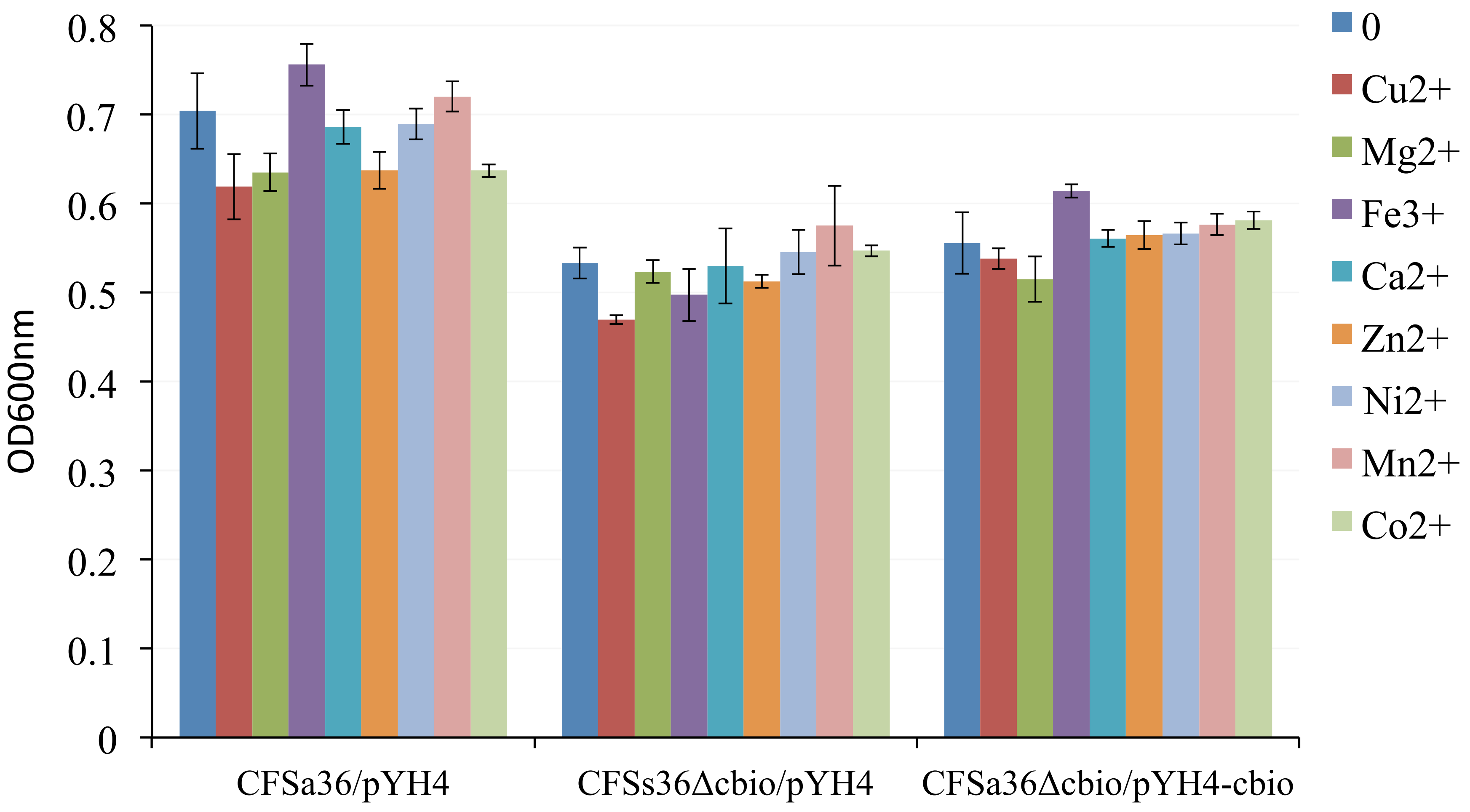
0.8
0.7
0.6
0.5
0.4
0.3
0.2
0.1
0
OD600nm
CFSa36/pYH4
CFSs36Δcbio/pYH4
CFSa36Δcbio/pYH4-cbio
0
Cu2+
Mg2+
Fe3+
Ca2+
Zn2+
Ni2+
Mn2+
Co2+

Figure S3

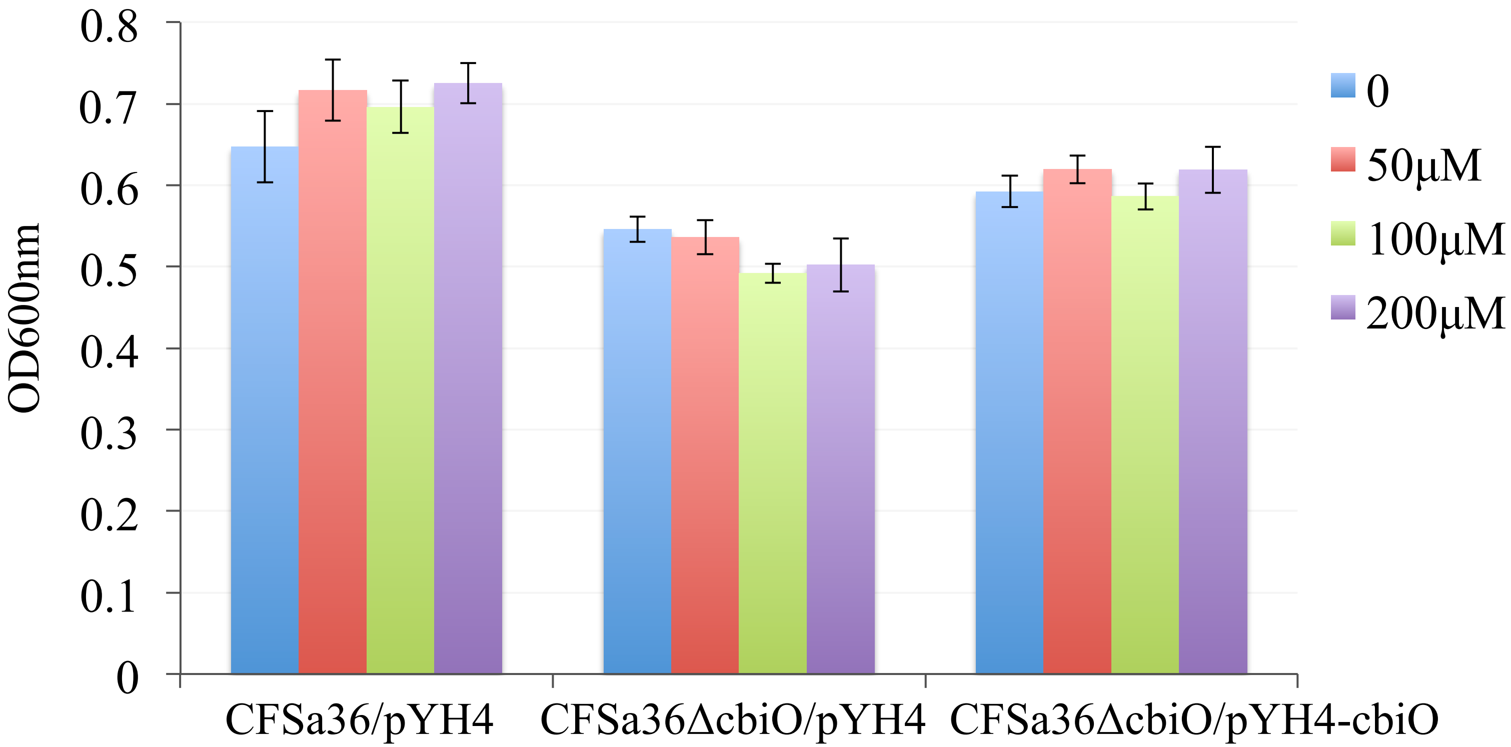
0.8
0.7
0.6
0.5
0.4
0.3
0.2
0.1
0
OD600nm
CFSa36/pYH4
CFSa36ΔcbiO/pYH4
CFSa36ΔcbiO/pYH4-cbiO
0
50μM
100μM
200μM

Figure S4
